# Supplementary material for: Added value of an open narrative in verbal autopsies: a mixed-methods evaluation from Malawi
Source: BMJ Paediatr Open. 2021 Feb 5;5(1):e000961. doi: 10.1136/bmjpo-2020-000961 (PMC7871275; doi:10.1136/bmjpo-2020-000961)
Supplement: Supplementary data [file bmjpo-2020-000961supp001.pdf]

Web-Appendix 1: Group discussion topic guide

1. What is your experience of conducting verbal autopsies?
2. Can you describe the typical interview process?
3. Thinking specifically about interviews which DID NOT have an open narrative:
    a. How did you establish a rapport with the respondents?
    b. Did you feel the quality of the responses was accurate?
    c. Were there any issues in conducting the interviews?
4. Now thinking specifically about the interviews which DID have an open narrative:
    a. How did you establish a rapport with the respondents?
    b. Did you feel the quality of the responses was accurate?
    c. Were there any issues in conducting the interviews?
5. Overall, what were the biggest challenges in conducting the verbal autopsies?
6. Overall, did you feel there were any positive aspects of conducting these interviews?
7. Did you feel the open narrative improved the interview process for respondents? And for you? Why / Why not?
8. If you could recommend a best practice for conducting verbal autopsies, what would it be? Why?

Web-appendix 2: Comparison of interviews which did not comply with the randomisation protocol

	**Interview conducted per protocol (n=2507)**	**Protocol violation**	
		No narrative group (n=22)	Narrative group (n=58)
**Respondent**			
Mother	1931 (77.0%)	19 (86.4%)	46 (79.3%)
Father	200 (8.0%)	2 (9.1%)	4 (6.9%)
Grandparent	266 (10.6%)	-	6 (10.3%)
Other	110 (4.4%)	1 (4.6%)	2 (3.5%)
**Socio-economic status by tercile**			
Tercile 1 (Lowest)	838 (33.4%)	6 (27.3%)	20 (34.5%)
Tercile 2 (Middle)	879 (35.1%)	8 (36.4%)	19 (32.8%)
Tercile 3 (Highest)	790 (31.5%)	8 (36.4%)	19 (32.8%)
**Child's age**			
Neonate (0-4 weeks)	1058 (41.9%)	7 (32.8%)	19 (32.8%)
Infant (5-52 weeks)	654 (26.4%)	10 (45.5%)	18 (31.0%)
Child (12-59 months)	795 (31.7%)	5 (22.7%)	21 (36.2%)
**Child's sex***			
Male	1326 (52.9%)	9 (40.1%)	24 (42.1%)
Female	1181 (47.1%)	13 (59.1%)	33 (57.9%)
**Location of death**			
Home	797 (31.8%)	8 (36.4%)	13 (22.4%)
Health centre	652 (26.0%)	5 (22.7%)	20 (34.5%)
MDH	616 (24.6%)	5 (22.7%)	12 (20.7%)
En-route to hospital	181 (7.2%)	-	6 (10.3%)
Other	261 (10.4%)	4 (18.2%)	7 (12.1%)
	**Mean (SD)**		
Delay between death and VA (weeks)	22.5 (15.5)	25.5 (14.9)	23.0 (14.3)

*One child in the non-compliant narrative group was missing a value for sex.

Web-Appendix 3: Comparison of randomised groups

	No Narrative N = 1265 (51%)	Narrative N = 1242 (49%)	Total N = 2507	*p-value*
**Respondent**				
Mother	985 (77.9%)	946 (76.2%)	1931 (77.0%)	
Father	95 (7.5%)	105 (8.5%)	200 (8.0%)	
Grandparent	125 (9.9%)	141 (11.4%)	266 (10.6%)	
Other	60 (4.7%)	50 (4.0%)	110 (4.4%)	0.400
**Socio-economic status by tercile**				
Tercile 1 (Lowest)	408 (32.3%)	430 (34.6%)	838 (33.4%)	
Tercile 2 (Middle)	459 (36.3%)	420 (33.8%)	879 (35.1%)	
Tercile 3 (Highest)	398 (31.5%)	392 (31.6%)	790 (31.5%)	0.343
**Child's age**				
Neonate (0-4 weeks)	505 (39.9%)	553 (44.5%)	1058 (41.9%)	
Infant (5-52 weeks)	352 (27.8%)	302 (24.3%)	654 (26.4%)	
Child (12-59 months)	408 (32.3%)	387 (31.2%)	795 (31.7%)	0.042
**Child's sex**				
Male	677 (53.5%)	649 (52.3%)	1326 (52.9%)	
Female	588 (46.5%)	593 (47.8%)	1181 (47.1%)	0.526
**Location of death**				
Home	408 (32.3%)	389 (31.3%)	797 (31.8%)	
Health centre	327 (26.9%)	325 (26.2%)	652 (26.0%)	
MDH	297 (23.5%)	319 (25.7%)	616 (24.6%)	
En route to hospital	78 (6.2%)	103 (8.3%)	181 (7.2%)	
Other	155 (12.3%)	106 (8.5%)	261 (10.4%)	0.008
	**Mean (SD)**	**Mean (SD)**		
Delay between death and VA (Weeks)	22.6 (15.5)	22.5 (15.4)	22.5 (15.5)	0.838
