# Supplementary material for: Added value of an open narrative in verbal autopsies: a mixed-methods evaluation from Malawi
Source: BMJ Paediatr Open. 2021 Feb 5;5(1):e000961. doi: 10.1136/bmjpo-2020-000961 (PMC7871275; doi:10.1136/bmjpo-2020-000961)
Supplement: Author's manuscript [file bmjpo-2020-000961.draft_revisions.pdf]

BMJ Paediatrics Open

BMJ Paediatrics Open is committed to open peer review. As part of this commitment we make the peer review history of every article we publish publicly available.

When an article is published we post the peer reviewers' comments and the authors' responses online. We also post the versions of the paper that were used during peer review. These are the versions that the peer review comments apply to.

The versions of the paper that follow are the versions that were submitted during the peer review process. They are not the versions of record or the final published versions. They should not be cited or distributed as the published version of this manuscript.

BMJ Paediatrics Open is an open access journal and the full, final, typeset and author-corrected version of record of the manuscript is available on our site with no access controls, subscription charges or pay-per-view fees (http://bmjpaedsopen.bmj.com).

If you have any questions on BMJ Paediatrics Open's open peer review process please email info.bmjpo@bmj.com

BMJ Paediatrics Open

The added value of an open narrative in verbal autopsies – a mixed-methods evaluation from Malawi

Journal:	*BMJ Paediatrics Open*
Manuscript ID	bmjpo-2020-000961
Article Type:	Original research
Date Submitted by the Author:	24-Nov-2020
Complete List of Authors:	Loh, Patricia; University College London, Institute for Global Health<br>Fottrell, Edward; University College London, Institute for Global Health<br>Beard, James; University College London, Institute for Global Health<br>Bar-Zeev, Naor; Malawi-Liverpool-Wellcome Trust Clinical Research Programme<br>Phiri, Tambosi; MaiMwana Project,<br>Banda, Masford; MaiMwana Project<br>Makwenda, Charles; Parent and Child Health Initiative<br>Bird, Jon; University of Bristol, Department of Computer Science<br>King, Carina; University College London, Institute for Global Health; Karolinska Institute, Department of Global Public Health
Keywords:	Data Collection, Ethics, Mortality, Qualitative research

SCHOLARONE™
Manuscripts

I, the Submitting Author has the right to grant and does grant on behalf of all authors of the Work (as defined in the below author licence), an exclusive licence and/or a non-exclusive licence for contributions from authors who are: i) UK Crown employees; ii) where BMJ has agreed a CC-BY licence shall apply, and/or iii) in accordance with the terms applicable for US Federal Government officers or employees acting as part of their official duties; on a worldwide, perpetual, irrevocable, royalty-free basis to BMJ Publishing Group Ltd ("BMJ") its licensees and where the relevant Journal is co-owned by BMJ to the co-owners of the Journal, to publish the Work in this journal and any other BMJ products and to exploit all rights, as set out in our licence.

The Submitting Author accepts and understands that any supply made under these terms is made by BMJ to the Submitting Author unless you are acting as an employee on behalf of your employer or a postgraduate student of an affiliated institution which is paying any applicable article publishing charge ("APC") for Open Access articles. Where the Submitting Author wishes to make the Work available on an Open Access basis (and intends to pay the relevant APC), the terms of reuse of such Open Access shall be governed by a Creative Commons licence – details of these licences and which Creative Commons licence will apply to this Work are set out in our licence referred to above.

Other than as permitted in any relevant BMJ Author's Self Archiving Policies, I confirm this Work has not been accepted for publication elsewhere, is not being considered for publication elsewhere and does not duplicate material already published. I confirm all authors consent to publication of this Work and authorise the granting of this licence.

Confidential: For Review Only

Title: The added value of an open narrative in verbal autopsies – a mixed-methods evaluation from Malawi

Authors: Patricia Loh[1], Edward Fottrell[1], James Beard[1], Naor Bar-Zeev[2,3], Tambosi Phiri[4], Masford Banda[4], Charles Makwenda[4], Jon Bird[5], Carina King[1,6*]

Affiliations:

1. Institute for Global Health, University College London, London, UK
2. Malawi Liverpool Wellcome Trust Clinical Research Programme, Blantyre, Malawi
3. International Vaccine Access Center, Department of International Health, Bloomberg School of Public Health, Johns Hopkins University, Baltimore MD, USA.
4. MaiMwana Project, Parent and Child Health Initiative, Lilongwe, Malawi
5. Department of Computer Science, University of Bristol, Bristol, UK
6. Department of Global Public Health, Karolinska Institutet, Stockholm, Sweden

***Corresponding author:**

Institute for Global Health, University College London, 30 Guildford Street, London WC1N 1EH.
Email: c.king@ucl.ac.uk

Word count: 3,621

Confidential: For Review Only

Abstract

Background

The WHO standardised verbal autopsy (VA) instrument includes closed questions, ascertaining signs and symptoms of illness preceding death, and an optional open narrative. As VA analyses increasingly use automated algorithms, inclusion of narratives should be justified. We evaluated the role of open narratives on VA processes, data quality and respondent emotion.

Methods

A mixed-methods analysis was conducted using VA data for children deaths (0–59 months), between 04/2013–11/2016 in Mchinji district, Malawi. Deaths were randomised at the point of interview to receive closed questions only or open narrative followed by closed questions. Upon concluding the VA, interviewers self-completed questions relating to respondent emotions. Logistic regression was conducted to determine associations with visible emotions during VAs. A group discussion with interviewers was conducted at the project end, to understand field experiences and explore future recommendations; data were coded using deductive themes.

Results

2509 VAs were included, with 49.8% (n=1341) randomised to open narratives. Narratives lasted a median of 7 minutes (range: 1–113). Interviewers described improved rapport and felt narratives improved data quality, although there was no difference in the proportion of deaths with an indeterminate cause (5.3% vs. 6.1%). The majority of respondents did not display visible emotions (81%). Those with a narrative had higher, but not statistically significant, odds of displaying emotion (aOR: 1.20; 95% CI: 0.98, 1.47). Factors associated with visible emotion were: infant deaths versus neonates; deaths at a health centre or on-route to hospital versus home; and higher socio-economic status. Non-parental respondents and increased time between death and interview were associated with lower odds of emotion.

Conclusion

Conducting an open narrative to build rapport, something valued by the interviewers, may outweigh additional time and emotional burdens. However, these burdens may be further justified if the quality and utility of information from the narrative was promoted through standardised recommendations.

Confidential: For Review Only

What is known about the topic

- Verbal autopsies are often conducted in contexts where civil registration systems are lacking or incomplete.
- There are several different tools for conducting these interviews, some containing an open narrative section, where respondents describe the events leading to a death in their own words.
- Automated methods for analysing verbal autopsies often do not use the data from narratives and therefore there should be a clear and justified reason for conducting this section of the interview.

What this study adds

- Data collectors reported the narratives as a way to build rapport with the respondents, and felt this improved their ability to collect better quality information.
- While respondents mostly did not show visible signs of emotions during interviews, this was more frequent but statistically non-significant, in those with a narrative.
- There may be a trade-off in the increased time and emotional burden of verbal autopsies which contain a narrative section, with the ability to establish a connection between data collectors and respondents.

Confidential: For Review Only

Background

A comprehensive civil registration and vital statistics (CRVS) system recording births and deaths provides a country with essential information to make informed decisions for country-specific priority-setting, and measure its progress towards the Sustainable Development Goals (1, 2). In the absence of functional CRVS structures, verbal autopsies (VA) can partly fill this gap as an interim mortality data collection instrument by providing cause-specific mortality estimates (3-5). These data can be used by governments, healthcare providers, researchers, donors and policy makers, who rely on accurate and comparable data over time to estimate burdens of diseases at population level, evaluate program implementation and complement routine administrative data (1, 6). A recent review supported the use of VA to identify vulnerable groups and health needs for effective resource allocation in humanitarian settings (7).

The VA process involves trained fieldworkers identifying and interviewing an appropriate respondent, usually a close relative or caregiver, for a given death (3). Events preceding the death are recorded using a survey with a pre-determined set of closed questions, which can be supplemented by a free-text open narrative designed to elicit the story in the respondent's own words of how the death occurred (8). Following this, a suspected cause of death is generally assigned through physician review, or through the automated application of statistical algorithms (e.g. InterVA or SmartVA) (3-5).

In 2006, up to 18 VA tools with varying combinations of closed questions and open narratives were reportedly being used in 13 countries (9). The World Health Organization (WHO) published the first iteration of a standardised VA methodology in 2007, with subsequent updates in 2012, 2014 and 2016 (8). The inclusion of an open narrative section remains recommended, but optional. The role of the narrative in physician-coded VAs has been likened to a medical history used by doctors to make diagnoses (10). It can also encourage interviewer-respondent rapport, providing respondents a more natural outlet to express themselves and recount events they feel were most relevant (11). The open narrative can also provide valuable information that standardised closed questions do not capture, such as cultural beliefs, adding context and holding authorities accountable to design interventions and services that are responsive to its people's needs (9, 12, 13). In contrast, it could be argued that such information could be better identified using structured social autopsy tools – a supplementary survey conducted specifically to identify non-medical causes of death (7, 14).

The emotional strain of a VA has been recently detailed in qualitative studies from Ghana,(15) Papua New Guinea,(16) and Nepal,(17) and fieldworkers from South Africa reported a higher likelihood of respondents becoming emotional during the open narrative compared to closed question sections of the interview (18). Furthermore, the potential for adverse effects of VA-induced distress on data quality, and the diagnostic influence this might have for assigning cause of death is important to understand (12, 19, 20).

This paper explores the role of the open narrative in the VA process, including its effects on interview procedures, data quality and respondent emotion. As the narrative potentially poses an additional burden on both respondents and interviewers, and VAs are increasingly analysed using automated algorithms that do not use these free-text responses, their inclusion in the VA process should be justified.

Confidential: For Review Only

Methods

We conducted a mixed-methods analysis of VA process data for deaths of children aged 0–59 months collected prospectively from April 2013 to November 2016, as part of the VacSurv Study in Mchinji District, Malawi (21). Mchinji is a rural agricultural district in the central region of Malawi, with a population of approximately 600000, under-5 mortality rate of 63 per 1000 livebirths and crude birth rate of 32.2 at the time of data collection (22).

Mortality Surveillance

Full details of the population surveillance system used by the VacSurv Study have been previously described (21, 23). Briefly, deaths in children aged 0–59 months, including stillbirths, were registered retrospectively from October 2011 to February 2012, and prospectively from March 2012 to June 2016. Births and deaths were reported by 1060 volunteer village informants who cumulatively covered the whole of Mchinji District, supervised by 50 enumerators and eight senior monitoring and evaluation officers (MEOs). Data were submitted using paper forms to the central office monthly where it was entered into a Microsoft Access database. Major errors in identification data (e.g. incompatible dates of birth and death) were sent back to the field for verification. All deaths in children under-5 years were extracted from the cleaned data, and pre-printed forms with a unique barcode containing the participant's study ID were generated.

Verbal Autopsies

Deaths were prospectively randomised at the point of interview to either: 1) closed questions only; or 2) open narrative followed by closed questions. Randomisation was programmed into the electronic data capture form (Open Data Kit software),(24) and the MEOs were informed of the allocation after the respondent had consented to the interview. The respondent was blinded to the randomisation procedure, but MEOs were unblinded to the purpose of randomisation. The open narrative was unstructured and MEOs could choose how they recorded the details, such as audio-recording and subsequent transcription, notes or direct transcription of the story during the interview. The closed questions were WHO's 2012 VA instrument,(25) translated into Chichewa.

Data Collection

The VAs were conducted at respondents' homes by nine different MEOs, each with five or more years' experience in conducting VAs. They underwent a one-week training where they collectively translated the WHO VA questionnaire, reviewed the study protocol including data collection using smartphones and strategies to conduct the interviews sensitively, and conducted supervised mock interviews.(12)

At the end of each VA, MEOs self-completed post-interview questions. MEOs were asked to document the respondents present, emotions during the interview and whether the interview needed to be paused as a result. Total VA interview duration was automatically captured on the smartphone, and MEOs noted the start and end time of the open narrative on the paper form.

Confidential: For Review Only

Closed questions were collected using ODK Collect on Android smartphones and narratives were submitted as written transcripts on the pre-printed forms. These were entered into a Microsoft Access database, and data were linked using the participants' study ID, then cleaned and processed.

Quantitative Analysis

Child characteristics and VA process data were described with proportions, means and standard deviations (SD) for normally distributed continuous data or medians and interquartile ranges (IQR) for asymmetrically distributed data. The types of emotion, ability to assign a cause of death, and duration of VA were compared between those with and without an open narrative, using t- and chi2 tests.

Cause of death was assigned using InterVA-4 (www.interva.net) based on closed question responses only; respondents had the option of answering with 'yes', 'no' or 'don't know'. InterVA uses a Bayesian model to calculate the likelihood of different causes of death based on positive ('yes') closed question responses only. The number of 'yes' answers and subsequently the ability to assign probable cause of death were used as a proxy measure of data quality from respondents. Emotion and interview duration were chosen as proxy indicators of burden for respondents and interviewers. Stillbirths were excluded from the analysis as we used a locally modified VA tool for these deaths.

The primary analysis was a per-protocol analysis, excluding interviews in which MEOs documented that a narrative was conducted when allocated to not include one, and vice versa. We compared respondent emotion during the interview between those with and without a narrative. A multivariable logistic regression was conducted, adjusted for potential confounders defined *a priori* as: main respondent, child's age and sex, location of death and socio-economic tercile. All analyses were conducted with Stata 15.0.

Qualitative Data Collection and Analysis

At study completion, a group discussion was held with the MEOs who conducted VAs during the project to gather their feedback on the utility of the open narrative and recommendations for VA procedures going-forward. This group discussion was led by the technical advisor (CK) in a private room within the office using a structured topic guide (**Web-Appendix 1**). The discussion was conducted in English, audio-recorded and transcribed verbatim. The data was then coded using the following deductive themes, based on the aim of the study: the interview process and procedures; perceived data quality; and emotions in VAs. The final analysis was shared with the MEOs, after triangulation with the quantitative analysis to check for interpretation.

Patient and Public Involvement

Prior to community verbal autopsies beginning, the overall VacSurv Study protocol was presented to the District Executive Committee and District Health Management team in Mchinji for input and approval. Extensive community engagement was conducted before data collection, and continued throughout the study, through village level key informant volunteers, area development committees and radio jingles. Community consent from traditional leaders was sought during study introduction.

Confidential: For Review Only

Ethics

Verbal informed consent was obtained for all VA interviews, and written consent for the group discussion participants. The study was approved by the National Health Sciences Research Ethics Committee in Malawi [#837], London School of Hygiene and Tropical Medicine, UK [#6047] and Centers for Disease Control and Prevention, USA [#6268].

Results

A total of 3623 under-5 deaths were reported during the study period, and 2509 were included in the analysis (**Figure 1**). Overall, 50.2% (n=1352) were allocated to no narrative and 49.8% (n=1341) to have an open narrative, with 95% and 94% of VAs conducted per-protocol in each arm. Balance in the randomisation was achieved for respondent type, socio-economic status, child sex and time since the death. However more open narrative interviews were conducted for neonates (44.5% versus 39.9%, *p*-value=0.042) and location of death differed between the two groups (**Web-Appendix 2**). Of the deaths, 41.9% were neonates, 52.9% were male and 31.8% occurred at home (**Web-Appendix 2**). Primary respondents were mainly mothers (77.0%, n=1931), followed by grandparents (10.6%, n=266) and fathers (8.0%, n=200). The mean time between death and verbal autopsy was 22.5 weeks (range: 1–52 weeks). We present the quantitative and qualitative results together under the following themes: VA processes, data quality and emotions.

VA Processes and Procedures

Open narratives took a median of 7 minutes (range: 1–113 minutes) and closed questions took 17.5 minutes (range: 6–164 minutes). Overall, interviews which included narratives took longer to complete, with 32.3% taking longer than 30 minutes compared to only 5.2% of those without a narrative (*p*-value < 0.001), with the accompanying closed questions correspondingly taking longer on average to complete (**Table 1**).

From the group discussion, MEOs reported narratives generally taking between 3 and 15 minutes. A key factor in the duration of these was the respondent and whether they were capable and willing to respond. Respondents who were keen to relay their story were reported to do so without prompting, including in interviews randomised to not include a narrative. Conversely respondents who were hard to engage in interviews with a narrative were also reported.

"My experience has been that after getting consent sometimes a respondent starts to recount before you ask, so you don't interrupt, you just listen. But because your phone has asked you not to take an open history, you don't take notes on that, you just go straight to the questions." (MEO 8)

"And you can see that there were some open histories that were very short, maybe just 2 minutes [general agreement]. You just know that the respondent was not ready to give you information. It happened like that." (MEO 2)

Confidential: For Review Only

However, in general the MEOs agreed that the main difference in interviews with and without open narratives was the time taken. Not taken into consideration in the quantitative measures of duration was the time to identify appropriate respondents before an interview could start. This could involve multiple visits to a respondent's household before an appropriate respondent could be located (e.g. mother or father), or needing to gain community trust to access the respondent.

"But some other times it may take even 10 minutes because these people know who you want to talk to you, but they are trying to shield them because they are not very sure at first what you've come to do." (MEO 1)

When asked what they would recommend as the best VA procedure, there was a consensus that both the open narrative and closed questions were important and should be included: *"The best way is the one which has the open history, that way you have the full explanation."* (MEO 7)

Data Quality

Based on InterVA analysis of closed questions, 94.3% of deaths had a cause of death assigned; there was no difference between those with and without an open narrative (94.7% versus 93.9%, *p*-value=0.404). Comparing the number of positive responses in the closed questions found no differences with a mean of 22.4, 21.4 and 21.8 "yes" answers for neonates, infant and child VA interviews (**Table 2**). The addition of the open narrative was not associated with respondents expressing a desire to know or suggest a potential cause of death.

There was consensus from the MEOs that data collected was of better quality when they conducted an open narrative. The first reason was that they effectively asked the questions twice, once as the narrative and then a second time in the closed questions, enabling them to cross-check responses. Secondly, MEOs reported respondents being more comfortable narrating a story than responding to "yes/no" questions.

"I have that feeling that, without the open history, the quality is compromised. Because it's like the recall system, the set-up of the brain of the respondent, is disturbed by question time and again. Unlike when he or she is free to express everything from her memory, it happens to be good quality data […] I think that open history gives a respondent a feeling that you are really concerned, because you take a lot of time to listen to him or her." (MEO 8)

While only 28.2% of respondents were recorded as providing a cause of death (**Table 2**), the MEOs noted that caregivers would often give a reason for their child's death – especially if they had sought care. However, they also noted that cause of death was not limited to medical reasons:

"In their narrations, they will tell you the cause, 'yes this baby was suffering from malaria, but we think this baby died because they delayed in referring us to a health centre'. Maybe in the most remote areas there was no ambulance, they were told to come to the [town] but the ambulance was not available. They were told to look for their own transport to the [town]. So they will tell you those ones as reasons, not the actual sickness of the baby." (MEO 4)

Confidential: For Review Only

Emotion

In the majority of interviews, respondents did not display visible signs of emotion (81%), with similar proportions between those with and without an open narrative (79.7% versus 82.4%, *p*-value=0.089). Of those who were recorded as showing signs of emotions, 3.4% cried, 26.7% had a long silence and 69.9% displayed other signs of emotion – over half of these interviews needed to be paused once or more (**Table 2**).

Table 3 shows the logistic regression for respondent emotion. While having an open narrative was associated with 20% (aOR: 1.20; 95% CI: 0.98, 1.47) higher likelihood of the respondent becoming emotional during the interview; this was not statistically significant but may be pragmatically relevant. Factors associated with lower odds of becoming emotional during the VA interview included: non-parental respondents and increased time between the death and interview (2% lower odds for each week passed). Factors associated with increased odds of visible displays of emotion include: deaths amongst infants compared to neonates (aOR: 1.42; 95% CI: 1.09, 1.85); the death occurring at a health centre (aOR: 1.36; 95% CI: 1.04, 1.77) or en-route to hospital (aOR: 1.49; 95% CI: 1.00, 2.22); and being in the middle (aOR 1.52; 95% CI: 1.17, 1.97) or highest wealth tercile (aOR: 1.49; 95% CI: 1.13, 1.95).

The emotion of respondents was not directly raised by the MEOs during the discussion; however, they noted a key challenge in conducting the VAs as being unable to help respondents or feeling hopeless when respondents related their stories. They raised specific examples around HIV positive respondents seeking advice or requests for referrals of malnourished children to NGO programmes.

"A challenge, in a nut shell, was not being able assist where questions were raised. You have raised questions to them. In the end they raise questions to you, that need action, for you to not be able to do anything. That was a big challenge and a let-down." (MEO 4)

The MEOs raised the fact that the VA process is emotional from the interviewer's perspective, as well as the respondent, with many of the MEOs also having families and young children which can relate to the narrative.

"The verbal autopsies are not easy to be carried as they involve or concern somebody who has lost life, so it's always emotional between the interviewer and the interviewee" (MEO 2)

Discussion

Using a mixed-methods analysis of VA process data amongst children under-five in Malawi, we explored the role of open narratives on the interview process, data quality and respondents' emotions. As expected, free-text narratives increased the duration of the VA interview but did not impact on the ability of a Bayesian algorithm to assign a cause of death - the proxy we used for data quality. The interviewers considered the open narrative useful in building rapport with respondents, agreeing with previously reported experiences, (11, 26) and believed it subsequently improved the VA data. However, respondents with an open narrative displayed visible emotions more frequently when compared to those without, even if visible respondent emotion was relatively uncommon.

https://mc.manuscriptcentral.com/bmjpo

Confidential: For Review Only

Although previous studies have observed VA-induced emotional stress amongst respondents, (16-18, 27) exploring characteristics of both the respondent and deceased showed interesting associations with visible emotion. Firstly, respondents were more likely to show emotion if they were of a higher socio-economic status. One hypothesis could be that under-five deaths are more common amongst lower socio-economic households; (28) specifically in Malawi, a study reported an under-five mortality of 52/1000 livebirths in the highest wealth group, compared to 69/1000 in middle and low wealth groups (29). The 'unexpectedness' of deaths amongst children has been found to be associated with increased parental grief previously (30). Therefore, as these are rarer events in families which are less underprivileged, with fewer perceived barriers to healthcare and prevention, this may affect respondent emotion.

We observed that deaths occurring at health centres or en-route to hospital was associated with increased emotion. This may reflect respondents' perception or experience of poor quality of care, resulting in frustrations at system failures and delays in referrals and receiving care. This was echoed by the MEOs, and prior data from this setting,(12) who described respondents attributing deaths to non-medical causes. Deficiencies in Malawian healthcare facilities' ability to deliver quality maternal, newborn and child care, have been found,(31) and modelled estimates suggest that poor quality maternal and newborn care result in considerable preventable mortality (32). Caregiver frustration with healthcare provision and challenges in reaching referral facilities is therefore understandable.

Although the MEOs perceived better rapport and improved data quality from VAs with open narratives, we did not observe any differences in the number of "yes" responses and the subsequent proportion of VAs with an assigned cause of death. Earlier findings from Malawi showed limited advantage in including open narratives to assign cause of death (12), however in this case it is hard to know whether individual answers would have been different. The added diagnostic value of the free-text narratives was also examined by Rankin et al., (19) who reported that the addition of the narrative did not explain discrepancies in diagnoses between physician and InterVA analyses. This could be due to narratives capturing indicators which are included in closed questions. A key principle in research ethics is to avoid intrusions;(33) therefore, if narrative data is not intended for analysis and does not appear to have any influence on data quality, documenting these data may pose an unnecessary burden.

A key limitation of our study was our reliance on interviewer-observed signs of respondent emotion. The MEO self-completed post-VA questionnaire may have suffered from the subjective nature of emotion and possible cultural norms of private bereavement. Including questions on respondent-reported emotional distress and interviewer self-reported emotion may have provided richer information. Secondly, it is likely that protocol violations occurred, as MEOs reported respondents being unwilling or unable to fully engage in the open narrative, and conversely narrating the story of their child's death without prompt. This is not unlike the reluctance observed in VA respondents in rural Ghana who occasionally denied interviews due to grief (15). While we planned a per-protocol analysis, we were unable to fully adjust for these violations in the quantitative analysis, and our results may therefore more closely reflect intention to treat. Finally, the group discussion with the MEOs was led by the technical advisor, possibly leading to social-desirability bias limiting their willingness to highlight concerns or deviations from the protocol.

https://mc.manuscriptcentral.com/bmjpo

Confidential: For Review Only

Conclusion

As far as we are aware, this is the first study to report the effect of open narratives during VA interviews on respondent emotion. Evidence from this large-scale evaluation suggests that open narratives do not necessarily affect data quality, but play a role in establishing rapport, which was clearly valued by interviewers. From the interviewer perspective, conducting an open narrative at the start of the VA may therefore outweigh the additional time burden and the slight increase in respondents becoming emotional. Any undue burden associated with having an open narrative would be further justified if the quality and utility of information from the narrative can be guaranteed. We would therefore recommend guidance from leading bodies, such as the WHO VA Reference Group, for a more standardised approach to record and analyse free-text narratives with a view to reducing bias introduced by those involved during the process such as interviewers, transcribers and reviewers. We would also support longer waiting periods between death and time of interview, so long as accurate recall is not negatively impacted, and the inclusion of wider non-parental family members to reduce the emotional burdens associated with the sensitive nature of discussing death.

Confidential: For Review Only

Declarations

Ethics approval and consent to participate

The study was approved by the National Health Sciences Research Ethics Committee in Malawi [#837], London School of Hygiene and Tropical Medicine, UK [#6047] and Centers for Disease Control and Prevention, USA [#6268]. Verbal informed consent was sought from respondents prior to starting the verbal autopsy interviews. Consent was documented in the electronic data capture form.

Consent for publication

Not applicable.

Availability of data and materials

Fully anonymised quantitative and qualitative datasets generated and analysed for the purpose of this study are available from the corresponding author, Dr. Carina King (c.king@ucl.ac.uk on reasonable request for research purposes only, following approval from the National Health Sciences Research Ethics Committee in Malawi, and study Principal Investigators.

Competing interests

The authors declare that they have no competing interests.

Funding

This VacSurv Project was funded by a Wellcome Trust Programme Grant (WT091909/B/10/Z) to Neil French, Nigel Cunliffe, Rob Heyderman; an investigator-initiated grant by GlaxoSmithKline Biologicals to Nigel Cunliffe, NBZ, Neil French; a Wellcome Trust Strategic Award to Anthony Costello (Number: 085417ma/Z/08/Z); and MLW core grant strategic award to Rob Heyderman.

Confidential: For Review Only

Author contributions

This study was conceived by PL, EF and CK, based on data from a larger research question conceived by NBZ, BJB, JB, EF and CK. The data was collected under the oversight of TP, CM, and MB. The quantitative data was analysed by PL and the qualitative data was coded and analysed by CK. The manuscript was written by PL, with significant input from CK. All authors read, commented and approved the manuscript.

Confidential: For Review Only

Acknowledgments

We would like to thank the communities and families participating in the study, the key informants for volunteering their time and the Traditional Authorities of Mchinji district and Chilumba, Karonga district for their support. We are very grateful for the hard work of our field and data staff. We thank the other VacSurv Consortium members: Neil French and Nigel Cunliffe (University of Liverpool); Rob Heydermann and Anthony Costello (University College London); Amelia Crampin (University of Glasgow); Osamu Nakagomi (University of Nagasaki); Jacqueline E. Tate and Umesh D. Parashar (Centers for Disease Control & Prevention, Atlanta, GA, USA). The findings and conclusions in this report are those of the authors and do not necessarily represent the official position of the US Centers for Disease Control and Prevention.

References

1. Thomas LM, D'Ambruoso L, Balabanova D. Verbal autopsy in health policy and systems: a literature review. BMJ Glob Health. 2018;3(2):e000639.
2. World health statistics 2018: monitoring health for the SDGs, sustainable development goals. Geneva; 2018.
3. D'Ambruoso L, Kahn K, Wagner RG, Twine R, Spies B, van der Merwe M, et al. Moving from medical to health systems classifications of deaths: extending verbal autopsy to collect information on the circumstances of mortality. Glob Health Res Policy. 2016;1:2.
4. Fottrell E, Byass P. Verbal Autopsy: Methods in Transition. Epidemiologic Reviews. 2010;32(1):38-55.
5. Murray CJL, Lozano R, Flaxman AD, Serina P, Phillips D, Stewart A, et al. Using verbal autopsy to measure causes of death: the comparative performance of existing methods. BMC Medicine. 2014;12(1):5.
6. AbouZahr C. Verbal autopsy: who needs it? Population Health Metrics. 2011;9(1):19.
7. Thomas LM, D'Ambruoso L, Balabanova D. Use of verbal autopsy and social autopsy in humanitarian crises. BMJ Glob Health. 2018;3(3):e000640.
8. Verbal autopsy standards: the 2016 WHO verbal autopsy instrument [online]. 2017.
9. Soleman N, Chandramohan D, Shibuya K. Verbal autopsy: current practices and challenges. Bulletin of the World Health Organization. 2006;84(3):239 - 45.
10. Reeves BC, Quigley M. A review of data-derived methods for assigning causes of death from verbal autopsy data. International Journal of Epidemiology. 1997;26(5):1080-9.
11. Lerer LB, Butchart A, Blanche MT. 'A bothersome death'—Narrative accounts of infant mortality in Cape Town, South Africa. Social Science & Medicine. 1995;40(7):945-53.
12. King C, Zamawe C, Banda M, Bar-Zeev N, Beard J, Bird J, et al. The quality and diagnostic value of open narratives in verbal autopsy: a mixed-methods analysis of partnered interviews from Malawi. BMC Medical Research Methodology. 2016;16(1):13.
13. Gouda HN, Flaxman AD, Brolan CE, Joshi R, Riley ID, AbouZahr C, et al. New challenges for verbal autopsy: Considering the ethical and social implications of verbal autopsy methods in routine health information systems. Soc Sci Med. 2017;184:65-74.
14. Moyer CA, Johnson C, Kaselitz E, Aborigo R. Using social autopsy to understand maternal, newborn, and child mortality in low-resource settings: a systematic review of the literature. Glob Health Action. 2017;10(1):1413917.
15. Aborigo RA, Allotey P, Tindana P, Azongo D, Debpuur C. Cultural imperatives and the ethics of verbal autopsies in rural Ghana. Global Health Action. 2013;6(1):18570.
16. Gouda HN, Kelly-Hanku A, Wilson L, Maraga S, Riley ID. "Whenever they cry, I cry with them": Reciprocal relationships and the role of ethics in a verbal autopsy study in Papua New Guinea. Soc Sci Med. 2016;163:1-9.
17. Morrison J, Fottrell E, Budhatokhi B, Bird J, Basnet M, Manandhar M, et al. Applying a Public Health Ethics Framework to Consider Scaled-Up Verbal Autopsy and Verbal Autopsy with Immediate Disclosure of Cause of Death in Rural Nepal. Public Health Ethics. 2018;11(3):293-310.
18. Bird J, Byass P, Kahn K, Mee P, Fottrell E. A matter of life and death: practical and ethical constraints in the development of a mobile verbal autopsy tool. Proceedings of the SIGCHI Conference on Human Factors in Computing Systems; Paris, France: Association for Computing Machinery; 2013. p. 1489–98.
19. Rankin JC, Lorenz E, Neuhann F, Yé M, Sié A, Becher H, et al. Exploring the role narrative free-text plays in discrepancies between physician coding and the InterVA regarding determination of malaria as cause of death, in a malaria holo-endemic region. Malaria Journal. 2012;11(1):51.
20. Joshi R, Praveen D, Chow C, Neal B. Effects on the estimated cause-specific mortality fraction of providing physician reviewers with different formats of verbal autopsy data. Population Health Metrics. 2011;9(1):33.

21. Bar-Zeev N, Kapanda L, King C, Beard J, Phiri T, Mvula H, et al. Methods and challenges in measuring the impact of national pneumococcal and rotavirus vaccine introduction on morbidity and mortality in Malawi. Vaccine. 2015;33(23):2637-45.
22. National Statistical Office/Malawi, ICF. Malawi Demographic and Health Survey 2015-16. Zomba, Malawi: National Statistical Office and ICF; 2017.
23. King C, Beard J, Crampin AC, Costello A, Mwansambo C, Cunliffe NA, et al. Methodological challenges in measuring vaccine effectiveness using population cohorts in low resource settings. Vaccine. 2015;33(38):4748-55.
24. Hartung C, Lerer A, Anokwa Y, Tseng C, Brunette W, Borriello G. Open data kit: tools to build information services for developing regions. Proceedings of the 4th ACM/IEEE International Conference on Information and Communication Technologies and Development; London, United Kingdom. 2369236: ACM; 2010. p. 1-12.
25. Verbal autopsy standards: the 2012 WHO verbal autopsy instrument [online]. Geneva; 2012.
26. BAIRAGI R, BLACK R, BLACKER J, BOERMA T, DALESSANDRO U, DAVID P, et al. MEASUREMENT OF OVERALL AND CAUSE-SPECIFIC MORTALITY IN INFANTS AND CHILDREN-MEMORANDUM FROM A WHO UNICEF MEETING (REPRINTED FROM BULLETIN DE LORGANISATION MONDIALE DE LA SANTE, VOL 72, PG 707-713, 1994). BULLETIN OF THE WORLD HEALTH ORGANIZATION. 1995;73(2):149-56.
27. Chandramohan D, Soleman N, Shibuya K, Porter J. Editorial: Ethical issues in the application of verbal autopsies in mortality surveillance systems. Tropical Medicine & International Health. 2005;10(11):1087-9.
28. Van Malderen C, Van Oyen H, Speybroeck N. Contributing determinants of overall and wealth-related inequality in under-5 mortality in 13 African countries. Journal of Epidemiology and Community Health. 2013;67(8):667-76.
29. Van Malderen C, Amouzou A, Barros AJD, Masquelier B, Van Oyen H, Speybroeck N. Socioeconomic factors contributing to under-five mortality in sub-Saharan Africa: a decomposition analysis. BMC Public Health. 2019;19(1):760.
30. Wijngaards-de Meij L, Stroebe M, Schut H, Stroebe W, van den Bout J, van der Heijden P, et al. Couples at Risk Following the Death of Their Child: Predictors of Grief Versus Depression. Journal of Consulting and Clinical Psychology. 2005;73(4):617-23.
31. Smith H, Asfaw AG, Aung KM, Chikoti L, Mgawadere F, d'Aquino L, et al. Implementing the WHO integrated tool to assess quality of care for mothers, newborns and children: results and lessons learnt from five districts in Malawi. BMC Pregnancy and Childbirth. 2017;17(1):271.
32. Chou VB, Walker N, Kanyangarara M. Estimating the global impact of poor quality of care on maternal and neonatal outcomes in 81 low- and middle-income countries: A modeling study. PLoS medicine. 2019;16(12):e1002990-e.
33. Vanclay F, Baines JT, Taylor CN. Principles for ethical research involving humans: ethical professional practice in impact assessment Part I. Impact Assessment and Project Appraisal. 2013;31(4):243-53.

Confidential: For Review Only

Figure 1: Verbal autopsy inclusion (CONSORT diagram)

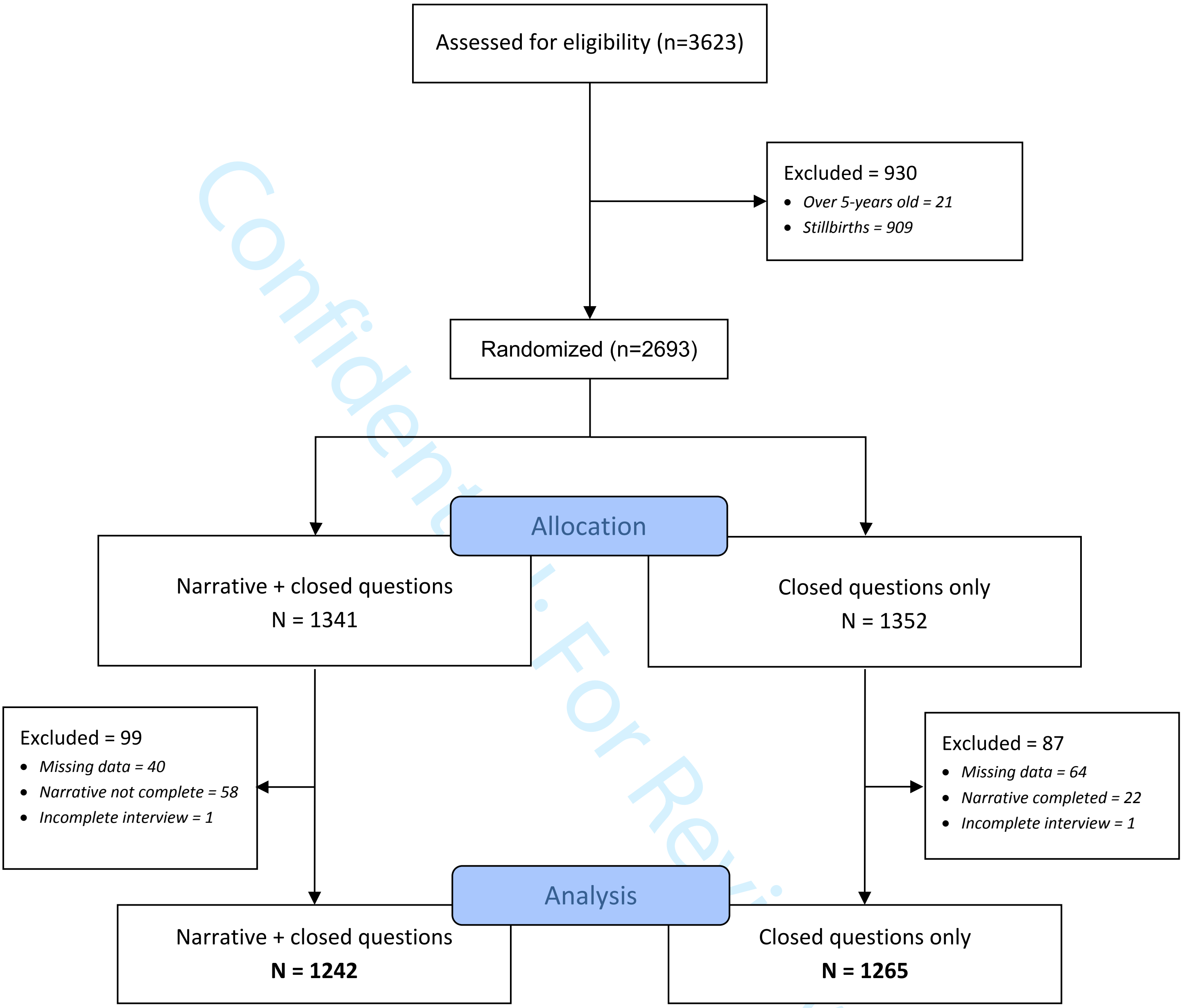

Confidential: For Review Only

Table 1: Description of VA duration.

	No Narrative N = 1265	Narrative N = 1242	*p-value*
**Total VA duration (Minutes)**			
< 10	259 (20.5%)	33 (2.7%)	
11 – 20	663 (52.4%)	381 (30.7)	
21 – 30	252 (19.9%)	422 (34.0%)	
> 30	66 (5.2%)	401 (32.3%)	
Missing	25 (2.0%)	5 (0.4%)	<0.001
**Closed question duration (Minutes)**			
Min – Max	6 – 134	6 – 164	
Median (IQR)	15.0 (9.7)	19.8 (9.9)	<0.001
**Narrative duration (Minutes)**			
Min – Max		1 – 113	
Median (IQR)		7.0 (5.0)	

Confidential: For Review Only

Table 2: Description of respondent emotions and VA quality indicators, between interviews with and without open narratives.

	No Narrative n (%)	Narrative n (%)	Total n (%)	*p-value*
**Respondent displayed visible emotion**				
No	1042 (82.4%)	990 (79.7%)	2032 (81.0%)	
Yes	223 (17.6%)	252 (20.3%)	475 (19.0%)	0.089
***Type of emotion displayed during interview**				
Crying	4 (1.8%)	12 (4.8%)	18 (3.4%)	
Long silence	59 (26.5%)	68 (27.0%)	127 (26.7%)	
Other signs of emotion	160 (71.8%)	172 (68.2%)	332 (69.9%)	0.191
***Interview paused due to respondent becoming too emotional**				
No	89 (39.9%)	117 (46.4%)	206 (43.4%)	
Yes – Once	31 (13.9%)	41 (16.3%)	72 (15.2%)	
Yes – More than once	103 (46.2%)	94 (37.3%)	197 (41.5%)	0.146
**Respondent expressed desire to know the cause of death**				
No	1235 (97.6%)	1216 (97.9%)	2451 (97.8%)	
Yes	30 (2.4%)	26 (2.1%)	56 (2.2%)	0.638
**Respondent suggested potential cause of death**				
No	909 (71.9%)	890 (71.7%)	1799 (71.8%)	
Yes	356 (28.1%)	352 (28.3%)	708 (28.2%)	0.912
**Inter-VA able to assign cause of death**				
Indeterminate	77 (6.1%)	66 (5.3%)	143 (5.7%)	
Determinate	1188 (93.9%)	1176 (94.7%)	2364 (94.3%)	0.404
**Number of "Yes" responses to closed questions****	**Mean (SD)**			
Neonates	22.6 (5.3)	22.2 (5.3)	22.4 (5.3)	0.297
Infants	21.5 (6.8)	21.3 (7.3)	21.4 (7.0)	0.658
Child	22.2 (8.2)	21.3 (8.1)	21.8 (8.1)	0.122

*Questions only asked for respondents who had a visible display of emotion (n=475)

**Different numbers of questions are asked for different age groups

For peer review only

Table 3: Logistic regression exploring associations between respondent and child characteristics and emotions during VA.

Visible emotion due to open narrative			
**Descriptors**		**aOR* (95% CI)**	***p-value***
**Open narrative**	No	1.00	
	Yes	1.20 (0.98, 1.47)	0.084
**Respondent**	Mother	1.00	
	Father	0.72 (0.49, 1.07)	0.102
	Grandparent	0.23 (0.13, 0.39)	<0.001
	Others	0.04 (0.01, 0.28)	0.001
**Child's age**	Neonate	1.00	
	Infant	1.42 (1.09, 1.85)	0.010
	Child under-5	1.21 (0.86, 1.69)	0.274
**Child's sex**	Male	1.00	
	Female	0.99 (0.80, 1.22)	0.920
**Location of death**	Home	1.00	
	Health centre	1.36 (1.04, 1.77)	0.023
	MDH	0.96 (0.72, 1.27)	0.753
	En route to hospital	1.49 (1.00, 2.22)	0.049
	Other	0.38 (0.23, 0.64)	<0.001
**Socio-economic status by tercile**	Tercile 1 (Lowest)	1.00	
	Tercile 2 (Middle)	1.52 (1.17, 1.97)	0.002
	Tercile 3 (Highest)	1.49 (1.13, 1.95)	0.004
**Delay between death & VA (Weeks)**		0.98 (0.98, 0.99)	0.002

*All variables presented were included in the adjusted analysis

Confidential: For Review Only

Web-Appendix 1: Group discussion topic guide

1. What is your experience of conducting verbal autopsies?
2. Can you describe the typical interview process?
3. Thinking specifically about interviews which DID NOT have an open narrative:
    a. How did you establish a rapport with the respondents?
    b. Did you feel the quality of the responses was accurate?
    c. Were there any issues in conducting the interviews?
4. Now thinking specifically about the interviews which DID have an open narrative:
    a. How did you establish a rapport with the respondents?
    b. Did you feel the quality of the responses was accurate?
    c. Were there any issues in conducting the interviews?
5. Overall, what were the biggest challenges in conducting the verbal autopsies?
6. Overall, did you feel there were any positive aspects of conducting these interviews?
7. Did you feel the open narrative improved the interview process for respondents? And for you? Why / Why not?
8. If you could recommend a best practice for conducting verbal autopsies, what would it be? Why?

Confidential: For Review Only

Web-Appendix 2: Comparison of randomised groups

	No Narrative N = 1265 (51%)	Narrative N = 1242 (49%)	Total N = 2507	*p-value*
**Respondent**				
Mother	985 (77.9%)	946 (76.2%)	1931 (77.0%)	
Father	95 (7.5%)	105 (8.5%)	200 (8.0%)	
Grandparent	125 (9.9%)	141 (11.4%)	266 (10.6%)	
Other	60 (4.7%)	50 (4.0%)	110 (4.4%)	0.400
**Socio-economic status by tercile**				
Tercile 1 (Lowest)	408 (32.3%)	430 (34.6%)	838 (33.4%)	
Tercile 2 (Middle)	459 (36.3%)	420 (33.8%)	879 (35.1%)	
Tercile 3 (Highest)	398 (31.5%)	392 (31.6%)	790 (31.5%)	0.343
**Child's age**				
Neonate	505 (39.9%)	553 (44.5%)	1058 (41.9%)	
Infant	352 (27.8%)	302 (24.3%)	654 (26.4%)	
Child under-5	408 (32.3%)	387 (31.2%)	795 (31.7%)	0.042
**Child's sex**				
Male	677 (53.5%)	649 (52.3%)	1326 (52.9%)	
Female	588 (46.5%)	593 (47.8%)	1181 (47.1%)	0.526
**Location of death**				
Home	408 (32.3%)	389 (31.3%)	797 (31.8%)	
Health centre	327 (26.9%)	325 (26.2%)	652 (26.0%)	
MDH	297 (23.5%)	319 (25.7%)	616 (24.6%)	
En route to hospital	78 (6.2%)	103 (8.3%)	181 (7.2%)	
Other	155 (12.3%)	106 (8.5%)	261 (10.4%)	0.008
	**Mean (SD)**	**Mean (SD)**		
Delay between death and VA (Weeks)	22.6 (15.5)	22.5 (15.4)	22.5 (15.5)	0.838

Confidential: For Review Only

BMJ Paediatrics Open

The added value of an open narrative in verbal autopsies – a mixed-methods evaluation from Malawi

Journal:	*BMJ Paediatrics Open*
Manuscript ID	bmjpo-2020-000961.R1
Article Type:	Original research
Date Submitted by the Author:	19-Jan-2021

Complete List of Authors:	Loh, Patricia; University College London, Institute for Global Health<br>Fottrell, Edward; University College London, Institute for Global Health<br>Beard, James; University College London, Institute for Global Health<br>Bar-Zeev, Naor; Malawi-Liverpool-Wellcome Trust Clinical Research Programme<br>Phiri, Tambosi; MaiMwana Project,<br>Banda, Masford; MaiMwana Project<br>Makwenda, Charles; Parent and Child Health Initiative<br>Bird, Jon; University of Bristol, Department of Computer Science<br>King, Carina; University College London, Institute for Global Health; Karolinska Institute, Department of Global Public Health

Keywords:	Data Collection, Ethics, Mortality, Qualitative research

SCHOLARONE™
Manuscripts

I, the Submitting Author has the right to grant and does grant on behalf of all authors of the Work (as defined in the below author licence), an exclusive licence and/or a non-exclusive licence for contributions from authors who are: i) UK Crown employees; ii) where BMJ has agreed a CC-BY licence shall apply, and/or iii) in accordance with the terms applicable for US Federal Government officers or employees acting as part of their official duties; on a worldwide, perpetual, irrevocable, royalty-free basis to BMJ Publishing Group Ltd ("BMJ") its licensees and where the relevant Journal is co-owned by BMJ to the co-owners of the Journal, to publish the Work in this journal and any other BMJ products and to exploit all rights, as set out in our licence.

The Submitting Author accepts and understands that any supply made under these terms is made by BMJ to the Submitting Author unless you are acting as an employee on behalf of your employer or a postgraduate student of an affiliated institution which is paying any applicable article publishing charge ("APC") for Open Access articles. Where the Submitting Author wishes to make the Work available on an Open Access basis (and intends to pay the relevant APC), the terms of reuse of such Open Access shall be governed by a Creative Commons licence – details of these licences and which Creative Commons licence will apply to this Work are set out in our licence referred to above.

Other than as permitted in any relevant BMJ Author's Self Archiving Policies, I confirm this Work has not been accepted for publication elsewhere, is not being considered for publication elsewhere and does not duplicate material already published. I confirm all authors consent to publication of this Work and authorise the granting of this licence.

Confidential: For Review Only

Title: The added value of an open narrative in verbal autopsies – a mixed-methods evaluation from Malawi

Authors: Patricia Loh[1], Edward Fottrell[1], James Beard[1], Naor Bar-Zeev[2,3], Tambosi Phiri[4], Masford Banda[4], Charles Makwenda[4], Jon Bird[5], Carina King[1,6*]

Affiliations:

1. Institute for Global Health, University College London, London, UK
2. Malawi Liverpool Wellcome Trust Clinical Research Programme, Blantyre, Malawi
3. International Vaccine Access Center, Department of International Health, Bloomberg School of Public Health, Johns Hopkins University, Baltimore MD, USA.
4. MaiMwana Project, Parent and Child Health Initiative, Lilongwe, Malawi
5. Department of Computer Science, University of Bristol, Bristol, UK
6. Department of Global Public Health, Karolinska Institutet, Stockholm, Sweden

***Corresponding author:**

Institute for Global Health, University College London, 30 Guildford Street, London WC1N 1EH.
Email: c.king@ucl.ac.uk

Word count: 4,122

Confidential: For Review Only

Abstract

Background

The WHO standardised verbal autopsy (VA) instrument includes closed questions, ascertaining signs and symptoms of illness preceding death, and an optional open narrative. As VA analyses increasingly use automated algorithms, inclusion of narratives should be justified. We evaluated the role of open narratives on VA processes, data quality and respondent's emotional stress.

Methods

A mixed-methods analysis was conducted using VA data for child deaths (0–59 months), between 04/2013–11/2016 in Mchinji district, Malawi. Deaths were prospectively randomised to receive closed questions only or open narrative followed by closed questions. Upon concluding the VA, interviewers self-completed questions on respondents' emotional stress. Logistic regression was used to determine associations with visible emotional distress during VAs. A group discussion with interviewers was conducted at the project end, to understand field experiences and explore future recommendations; data were coded using deductive themes.

Results

2509 VAs were included, with 49.8% (n=1341) randomised to open narratives. Narratives lasted a median of 7 minutes (range: 1–113). Interviewers described improved rapport and felt narratives improved data quality, although there was no difference in the proportion of deaths with an indeterminate cause using an automated algorithm (5.3% vs. 6.1%). The majority of respondents did not display visible emotional stress (81%). Those with a narrative had higher, but not statistically significant, odds of emotional distress (aOR: 1.20; 95% CI: 0.98, 1.47). Factors associated with emotional stress were: infant deaths versus neonates; deaths at a health centre or en-route to hospital versus home; and higher socio-economic status. Non-parental respondents and increased time between death and interview were associated with lower odds of emotional distress.

Conclusion

Conducting an open narrative may help build rapport, something valued by the interviewers. However, additional time and emotional burdens should be further justified, with quality and utility of narratives promoted through standardised recommendations.

Confidential: For Review Only

What is known about the topic

- Verbal autopsies are often conducted in contexts where civil registration systems are lacking or incomplete.
- There are several different tools for conducting these interviews, some containing an open narrative section, where respondents describe the events leading to a death in their own words.
- Automated methods for analysing verbal autopsies often do not use the data from narratives and therefore there should be a clear and justified reason for conducting this section of the interview.

What this study adds

- Data collectors reported the narratives as a way to build rapport with the respondents, and felt this improved their ability to collect better quality information.
- While respondents mostly did not show visible signs of emotional stress during interviews, this was more frequent but statistically non-significant, in those with a narrative.
- There may be a trade-off in the increased time and emotional burden of verbal autopsies which contain a narrative section, with the ability to establish a connection between data collectors and respondents.

Confidential: For Review Only

Background

A comprehensive civil registration and vital statistics (CRVS) system recording births and deaths provides a country with essential information to make informed decisions for country-specific priority-setting, and measure its progress towards the Sustainable Development Goals (1, 2). In the absence of functional CRVS structures, verbal autopsies (VA) can partly fill this gap as an interim mortality data collection instrument by providing cause-specific mortality estimates (3-5). These data can be used by governments, healthcare providers, researchers, donors and policy makers, who rely on accurate and comparable data over time to estimate burdens of diseases at population level, evaluate program implementation and complement routine administrative data (1, 6). A recent review supported the use of VA to identify vulnerable groups and health needs for effective resource allocation in humanitarian settings (7).

The VA process involves trained fieldworkers identifying and interviewing an appropriate respondent, usually a close relative or caregiver, for a given death. Events preceding the death are recorded using a survey with a pre-determined set of closed questions, which can be supplemented by a free-text open narrative designed to elicit the story in the respondent's own words of how the death occurred (8). Following this, a suspected cause of death is generally assigned through physician review, or through the automated application of statistical algorithms (e.g. InterVA or SmartVA) (3-5).

In 2006, up to 18 VA tools with varying combinations of closed questions and open narratives were reportedly being used in 13 countries (9). The World Health Organization (WHO) published the first iteration of a standardised VA methodology in 2007, with subsequent updates in 2012, 2014 and 2016 (8). The inclusion of an open narrative section remains recommended, but optional. The role of the narrative in physician-coded VAs has been likened to a medical history used by doctors to make diagnoses (10). It can also encourage interviewer-respondent rapport, providing respondents a more natural outlet to express themselves and recount events they feel were most relevant (11). The open narrative can also provide valuable information that standardised closed questions do not capture, such as cultural beliefs, adding context and holding authorities accountable to design interventions and services that are responsive to its people's needs (9, 12, 13). In contrast, it could be argued that such information could be better identified using structured social autopsy tools – a supplementary survey conducted specifically to identify non-medical causes of death (7, 14).

The emotional strain of a VA has been detailed in qualitative studies from Ghana,(15) Papua New Guinea,(16) and Nepal,(17) and fieldworkers from South Africa reported a higher likelihood of respondents becoming emotionally stressed during the open narrative compared to closed question sections of the interview (18). Furthermore, the potential for adverse effects of VA-induced distress on data quality, and the diagnostic influence this might have for assigning cause of death is important to understand (12, 19, 20).

This paper explores the role of the open narrative in the VA interview process, including its effects on procedures, data quality and emotional stress in respondents. Narratives potentially pose additional burdens on both respondents and interviewers, and as VAs are increasingly analysed using automated algorithms that do not use these free-text responses, their inclusion in the VA process should be justified.

Confidential: For Review Only

Methods

We conducted a mixed-methods analysis of VA process data for deaths of children aged 0–59 months collected prospectively from April 2013 to November 2016, as part of the VacSurv Study in Mchinji District, Malawi (21). Mchinji is a rural agricultural district in the central region of Malawi, with a population of approximately 600000, under-5 mortality rate of 63 per 1000 livebirths and crude birth rate of 32.2 at the time of data collection (22).

Mortality Surveillance

Full details of the population surveillance system used by the VacSurv Study have been previously described (21, 23). Briefly, deaths in children aged 0–59 months, including stillbirths, were registered retrospectively from October 2011 to February 2012, and prospectively from March 2012 to June 2016. Births and deaths were reported by 1060 volunteer village informants who cumulatively covered the whole of Mchinji District, supervised by 50 enumerators and eight senior monitoring and evaluation officers (MEOs). Data were submitted using paper forms to the central office monthly where it was entered into a Microsoft Access database. Major errors in identification data (e.g. incompatible dates of birth and death) were sent back to the field for verification. All deaths in children under-5 years were extracted from the cleaned data, and pre-printed forms with a unique barcode containing the participant's study ID were generated.

Verbal Autopsies

Deaths were prospectively randomised to one of two standard VA approaches: 1) closed questions only; or 2) open narrative followed by closed questions. Randomisation was programmed into the electronic data capture form (Open Data Kit software),(24) and the MEOs were informed of the allocation after the respondent had consented to the interview. The respondent was blinded to the randomisation procedure to minimise potential recall and volunteer biases, but MEOs were unblinded to the purpose of randomisation. The open narrative was unstructured and MEOs could choose how they recorded the details, such as audio-recording and subsequent transcription, notes or direct transcription of the story during the interview. The closed questions were WHO's 2012 VA instrument,(25) translated into Chichewa.

Data Collection

The VAs were conducted at respondents' homes by nine different MEOs. The MEOs were Malawian males, who resided in Mchinji district and had completed secondary education. Several hold diplomas in community mobilisation and social work. All had worked within the local communities for a minimum of five years before project commencement, and had conducted VAs previously. They underwent a one-week training, including: collective translation of the WHO VA questionnaire; study protocol including data collection using smartphones; developed Standard Operating Procedures (SOPs) for conducting the interviews sensitively; and supervised mock interviews.(12) The SOPs included identifying the main respondent, consent procedures, managing respondent distress and offering condolences.

Confidential: For Review Only

At the end of each VA, MEOs self-completed post-interview questions. MEOs were asked to document the respondents present, emotional stress during the interview and whether the interview needed to be paused as a result. Total VA interview duration was automatically captured on the smartphone, and MEOs noted the start and end time of the open narrative on the paper form.

Closed questions were collected using ODK Collect on Android smartphones and narratives were submitted as written transcripts on the pre-printed forms. These were entered into a Microsoft Access database, and data were linked using the participants' study ID, then cleaned and processed.

Quantitative Analysis

Child characteristics and VA process data were described with proportions, means and standard deviations (SD) for normally distributed continuous data or medians and interquartile ranges (IQR) for asymmetrically distributed data. Student's t- and chi2 test were applied to the comparisons of process data between those with and without narratives.

Cause of death was assigned using InterVA-4 (www.interva.net) based on closed question responses only; respondents had the option of answering with "yes", "no" or "don't know". InterVA uses a Bayesian model to assign the posterior probability of different causes of death based on positive ("yes") closed question responses only. The number of "yes" answers and subsequently the ability to assign probable cause of death were used as a proxy measure of data quality. Emotional distress and interview duration were chosen as proxy indicators of burden for respondents and interviewers. Stillbirths were excluded from the analysis as we used a locally modified VA tool for these deaths. The narratives were not used for assigning cause of death, and it was outside the scope of the study to validate or verify automated cause of death assignment.

The primary analysis was a per-protocol analysis (i.e. excluding interviews not conducted as allocated). This was chosen to examine the mechanism of narratives and the relationship to respondent distress, and not the process of recommending a narrative be done. We compared respondent emotional distress during the interview between those with and without a narrative. A multivariable logistic regression was conducted, adjusted for potential confounders defined *a priori* as: main respondent, child's age and sex, location of death and socio-economic tercile. All analyses were conducted with Stata 15.0.

Qualitative Data Collection and Analysis

At study completion, a group discussion was held with the MEOs who conducted VAs during the project to gather their feedback on the utility of the open narrative, their recommendations for future VA procedures and debrief on the emotional toll of administering VAs. . This group discussion was led by the technical advisor (CK) in a private room within the office using a structured topic guide (**Web-Appendix 1**). The discussion was conducted in English, audio-recorded and transcribed verbatim. The data was coded by CK using pen and paper, with pre-defined deductive themes using a thematic approach. The themes were based on the aim of the study: the interview process and procedures; perceived data quality; and emotional stress during VAs. The final interpretation was

Confidential: For Review Only

shared with the MEOs, after triangulation with the quantitative analysis to check that it corresponded with their experiences.

Patient and Public Involvement

Prior to community VAs beginning, the overall VacSurv Study protocol was presented to the District Executive Committee and District Health Management team in Mchinji for input and approval. Extensive community engagement was conducted before data collection, and continued throughout the study, through village level key informant volunteers, area development committees and radio jingles. Community consent from traditional leaders was sought during study introduction.

Ethics

Verbal informed consent was obtained for all VA interviews, and written consent for the group discussion participants. The study was approved by the National Health Sciences Research Ethics Committee in Malawi [#837], London School of Hygiene and Tropical Medicine, UK [#6047] and Centers for Disease Control and Prevention, USA [#6268].

Results

A total of 3623 under-5 deaths were reported during the study period, and 2507 were included in the analysis (**Figure 1**). Overall, 50.2% (n=1352) were allocated to no narrative and 49.8% (n=1341) to have an open narrative, with 95% and 94% of VAs conducted per-protocol in each arm (Web-Appendix 2). Balance in the randomisation was achieved for respondent type, socio-economic status, child sex and time since the death. However more open narrative interviews were conducted for neonates (44.5% versus 39.9%, p-value=0.042) and location of death differed between the two groups (**Web-Appendix 3**). Of the deaths, 41.9% were neonates, 52.9% were male and 31.8% occurred at home (**Web-Appendix 3**). Primary respondents were mainly mothers (77.0%, n=1931), followed by grandparents (10.6%, n=266) and fathers (8.0%, n=200). The mean time between death and verbal autopsy was 22.5 weeks (range: 1–52 weeks). We present the quantitative and qualitative results together under the following themes: VA processes, data quality and emotional stress.

VA Processes and Procedures

Open narratives took a median of 7 minutes (range: 1–113 minutes) and closed questions took 17.5 minutes (range: 6–164 minutes). Overall, interviews which included narratives took longer to complete, with 32.3% taking longer than 30 minutes compared to only 5.2% of those without a narrative (p-value < 0.001), with the accompanying closed questions correspondingly taking longer on average to complete (**Table 1**).

From the group discussion, MEOs reported narratives generally taking between 3 and 15 minutes. A key factor in the duration of these was the respondent and whether they were capable and willing to

https://mc.manuscriptcentral.com/bmjpo

Confidential: For Review Only

respond. Respondents who were keen to relay their story were reported to do so without prompting, including in interviews randomised to not include a narrative. Conversely respondents who were hard to engage in interviews with a narrative were also reported.

"My experience has been that after getting consent sometimes a respondent starts to recount before you ask, so you don't interrupt, you just listen. But because your phone has asked you not to take an open history, you don't take notes on that, you just go straight to the questions." (MEO 8)

"And you can see that there were some open histories that were very short, maybe just 2 minutes [general agreement]. You just know that the respondent was not ready to give you information. It happened like that." (MEO 2)

However, in general the MEOs agreed that the main difference in interviews with and without open narratives was the time taken. Not taken into consideration in the quantitative measures of duration was the time to identify appropriate respondents before an interview could start. This could involve multiple visits to a respondent's household before an appropriate respondent could be located (e.g. mother or father), or needing to gain community trust to access the respondent.

"But some other times it may take even 10 minutes because these people know who you want to talk to you, but they are trying to shield them because they are not very sure at first what you've come to do." (MEO 1)

When asked what they would recommend as the best VA procedure, there was a consensus that both the open narrative and closed questions were important and should be included: *"The best way is the one which has the open history, that way you have the full explanation."* (MEO 7)

Data Quality

Based on InterVA analysis of closed questions, 94.3% of deaths had a cause of death assigned; there was no difference between those with and without an open narrative (94.7% versus 93.9%, *p*-value=0.404). Comparing the number of positive responses in the closed questions found no differences with a mean of 22.4, 21.4 and 21.8 "yes" answers for neonates, infant and child VA interviews (**Table 2**). The addition of the open narrative was not associated with respondents expressing a desire to know or suggest a potential cause of death.

There was consensus from the MEOs that data collected was of better quality when they conducted an open narrative. The first reason was that they effectively asked the questions twice, once as the narrative and then a second time in the closed questions, enabling them to cross-check responses. Secondly, MEOs reported respondents being more comfortable narrating a story than responding to "yes/no" questions. Finally, they reported the information gained during the narrative helped them navigate through the closed questions and probe respondents for details in a more directed fashion.

"I have that feeling that, without the open history, the quality is compromised. Because it's like the recall system, the set-up of the brain of the respondent, is disturbed by questions time and again. Unlike when he or she is free to express everything from her memory, it happens to be good quality

Confidential: For Review Only

data […] I think that open history gives a respondent a feeling that you are really concerned, because you take a lot of time to listen to him or her." (MEO 8)

While only 28.2% of respondents were recorded as providing a cause of death (**Table 2**), the MEOs noted that caregivers would often give a reason for their child's death – especially if they had sought care. However, they also noted that cause of death was not limited to medical reasons:

"In their narrations, they will tell you the cause, 'yes this baby was suffering from malaria, but we think this baby died because they delayed in referring us to a health centre'. Maybe in the most remote areas there was no ambulance, they were told to come to the [town] but the ambulance was not available. They were told to look for their own transport to the [town]. So they will tell you those ones as reasons, not the actual sickness of the baby." (MEO 4)

Emotional stress

In the majority of interviews, respondents did not display visible signs of emotional distress (81%), with similar proportions between those with and without an open narrative (79.7% versus 82.4%, *p*-value=0.089). Of those who were recorded as showing signs of emotional distress, 3.4% cried, 26.7% had a long silence and 69.9% exhibited other signs of emotional distress – over half of these interviews needed to be paused once or more (**Table 2**).

Table 3 shows the logistic regression for respondent emotional distress. While having an open narrative was associated with 20% (aOR: 1.20; 95% CI: 0.98, 1.47) higher odds of the respondent becoming emotionally distressed during the interview; this was not statistically significant but may be pragmatically relevant. Factors associated with lower odds of becoming emotionally stressed during the VA interview included: non-parental respondents and increased time between the death and interview (2% lower odds for each week passed). Factors associated with increased odds of visible signs of emotional stress include: deaths amongst infants compared to neonates (aOR: 1.42; 95% CI: 1.09, 1.85); the death occurring at a health centre (aOR: 1.36; 95% CI: 1.04, 1.77) or en route to hospital (aOR: 1.49; 95% CI: 1.00, 2.22); and being in the middle (aOR 1.52; 95% CI: 1.17, 1.97) or highest wealth tercile (aOR: 1.49; 95% CI: 1.13, 1.95).

Respondents' emotional stress was not directly raised by the MEOs during the discussion; however, they noted a key challenge in conducting the VAs as being unable to help respondents or feeling hopeless when respondents related their stories. They raised specific examples around HIV positive respondents seeking advice or requests for referrals of malnourished children to NGO programmes.

"A challenge, in a nut shell, was not being able assist where questions were raised. You have raised questions to them. In the end they raise questions to you, that need action, for you to not be able to do anything. That was a big challenge and a let-down." (MEO 4)

The MEOs indicated that the VA process is similarly distressing for the interviewer, with many of the MEOs also having families and young children which can relate to the narrative.

"The verbal autopsies are not easy to be carried as they involve or concern somebody who has lost life, so it's always emotional between the interviewer and the interviewee" (MEO 2)

Confidential: For Review Only

Discussion

Using a mixed-methods analysis of VA process data amongst children under-five in Malawi, we explored the role of open narratives on the interview process, data quality and respondents' emotional stress. As expected, free-text narratives increased the duration of the VA interview but did not impact on the ability of a Bayesian algorithm to assign a cause of death - the proxy we used for data quality. The interviewers considered the open narrative useful in building rapport with respondents, agreeing with previously reported experiences,(11, 26) and believed it subsequently improved the VA data. However, respondents with an open narrative displayed emotional distress more frequently when compared to those without, even if visible relatively uncommon. While it was outside the scope of this study, further work is warranted from the respondent perspective; in particular, whether they value the space to narrate their stories, and how this balances with the emotional burden.

Although previous studies have observed VA-induced emotional stress amongst respondents,(16-18, 27) exploring characteristics of both the respondent and deceased showed interesting associations with emotional distress. Firstly, infant death was associated with increased emotional stress during VA compared to neonatal deaths. Grief is influenced by cultural constructs, and here cultural perceptions of child 'maturity' may be important. Studies from Tanzania and Ghana both point to norms around concealing mourning for young infants, in particular pregnancy loss (28, 29). Respondents from the higher socio-economic group had lower odds of observed emotional stress. Under-five deaths are more frequent amongst lower socio-economic households;(30) with an under-five mortality of 52/1000 livebirths in the highest wealth group, compared to 69/1000 in middle and low wealth groups in Malawi (31). The 'unexpectedness' of deaths amongst children has been associated with increased parental grief previously (32); and a study from South Africa reported that pressures of poverty can overshadow the grieving process (33). More understanding on how local contexts and mourning processes can affect the VA procedure, would be valuable.

We observed that deaths occurring at health centres or en route to hospital were associated with increased emotional stress. This may reflect respondents' perception or experience of poor quality of care, resulting in frustrations at system failures and delays in referrals and receiving care. This was echoed by the MEOs, and prior data from this setting,(12) who described respondents attributing deaths to non-medical causes. Deficiencies in Malawian healthcare facilities' ability to deliver quality maternal, newborn and child care have been reported,(34) and modelled estimates suggest that poor quality maternal and newborn care result in considerable preventable mortality (35). Caregiver frustration with healthcare provision and challenges in reaching referral facilities is therefore understandable.

Although the MEOs perceived better rapport and improved data quality from VAs with open narratives, we did not observe any differences in the number of "yes" responses and the subsequent proportion of VAs with an assigned cause of death. Earlier findings from Malawi showed limited advantage in including unstructured open narratives to assign cause of death. (12) While in this case it is hard to know whether individual answers would have been different, comparing cause of death distributions between those with and without narratives found no clear differences (data not shown), suggesting this was not the case. The added diagnostic value of free-text narratives has

Confidential: For Review Only

been examined previously (19), and found that the addition of the narrative did not explain discrepancies in diagnoses between physician and InterVA analyses. This could be due to narratives capturing indicators which are already included in closed questions. A key principle in research ethics is to avoid intrusions;(36) therefore, if narrative data is not intended for analysis and does not appear to have any influence on data quality, documenting these data may pose an unnecessary burden.

The main limitation of our study was our reliance on interviewer-observed signs of respondent emotion. The MEO self-completed post-VA questionnaire may have suffered from the subjective nature of expression and interpretation of emotion, and cultural norms of private bereavement. Grief after the death of an infant has also been described as a non-linear process, and influenced by gender (37). Including questions on both respondent-reported and interviewer self-reported emotional stress would have provided richer information. The MEOs also reported being upset by the VA interviews. A study from Mexico has developed a containment strategy to support the emotional health of data collectors conducting VAs, and going forward this should be considered (38).

It has been reported that women can face stigma and blame in child deaths (39). The power imbalance and gendered interviewer-interviewee dynamics present in this study may therefore have influenced mothers' emotional stress and willingness to freely discuss their child's death. These dynamics may also be present when multiple respondents were interviewed together e.g. a husband and wife. While we allowed the main respondent to decide who else was present for the VA, women may not have been empowered to exclude others from the process. The project conducted extensive community sensitisation through working with village leaders and key informants to gain respondent trust. However, the MEOs being local residents may also have inadvertently affected this, as Haws et. al, found interviewers from outside the community with good cultural insights may be more be trusted (29).

It is likely that undocumented protocol violations occurred, as MEOs reported respondents being unwilling or unable to fully engage in the open narrative, and conversely narrating the story of their child's death without prompt. This is not unlike the reluctance observed in VA respondents in rural Ghana who occasionally denied interviews due to grief (15). While we planned a per-protocol analysis, we were unable to fully adjust for these violations in the quantitative analysis, and our results may therefore more closely reflect intention-to-treat. Finally, the group discussion with the MEOs was led by the technical advisor, possibly leading to social-desirability bias limiting their willingness to highlight concerns or deviations from the protocol.

Conclusion

Evidence from this large-scale evaluation suggests that open narratives do not affect the ability of an automated algorithm to assign a cause of death, but play a valued role in establishing interviewer-interviewee rapport. From the interviewer perspective, good rapport as a result of conducting an open narrative at the start of the VA may outweigh the additional time burden and the slight increase in emotional stress amongst respondents. Any undue burden associated with having an open narrative would be further justified if the quality and utility of information can be guaranteed.

Confidential: For Review Only

We would therefore recommend guidance from leading bodies, such as the WHO VA Reference Group, for a more standardised approach to record and analyse free-text narratives. This is with a view to reducing bias introduced by those involved during the VA process, but should also take into account respondent perspective and preferences. We would also support longer waiting periods between death and time of interview, so long as accurate recall is not negatively impacted, and the inclusion of wider non-parental family members to reduce the emotional burdens associated with the sensitive nature of discussing death.

Confidential: For Review Only

Declarations

Ethics approval and consent to participate

The study was approved by the National Health Sciences Research Ethics Committee in Malawi [#837], London School of Hygiene and Tropical Medicine, UK [#6047] and Centers for Disease Control and Prevention, USA [#6268]. Verbal informed consent was sought from respondents prior to starting the verbal autopsy interviews. Consent was documented in the electronic data capture form.

Consent for publication

Not applicable.

Availability of data and materials

Fully anonymised quantitative and qualitative datasets generated and analysed for the purpose of this study are available from the corresponding author, Dr. Carina King (c.king@ucl.ac.uk on reasonable request for research purposes only, following approval from the National Health Sciences Research Ethics Committee in Malawi, and study Principal Investigators.

Competing interests

The authors declare that they have no competing interests.

Funding

This VacSurv Project was funded by a Wellcome Trust Programme Grant (WT091909/B/10/Z) to Neil French, Nigel Cunliffe, Rob Heyderman; an investigator-initiated grant by GlaxoSmithKline Biologicals to Nigel Cunliffe, NBZ, Neil French; a Wellcome Trust Strategic Award to Anthony Costello (Number: 085417ma/Z/08/Z); and MLW core grant strategic award to Rob Heyderman.

Confidential: For Review Only

421 **Author contributions**

422 This study was conceived by PL, EF and CK, based on data from a larger research question conceived
423 by NBZ, BJB, JB, EF and CK. The data was collected under the oversight of TP, CM, and MB. The
424 quantitative data was analysed by PL and the qualitative data was coded and analysed by CK. The
425 manuscript was written by PL, with significant input from CK. All authors read, commented and
426 approved the manuscript.

427

428 **Acknowledgments**

429 We would like to thank the communities and families participating in the study, the key informants
430 for volunteering their time and the Traditional Authorities of Mchinji district and Chilumba, Karonga
431 district for their support. We are very grateful for the hard work of our field and data staff. We
432 thank the other VacSurv Consortium members: Neil French and Nigel Cunliffe (University of
433 Liverpool); Rob Heydermann and Anthony Costello (University College London); Amelia Crampin
434 (University of Glasgow); Osamu Nakagomi (University of Nagasaki); Jacqueline E. Tate and Umesh D.
435 Parashar (Centers for Disease Control & Prevention, Atlanta, GA, USA). The findings and conclusions
436 in this report are those of the authors and do not necessarily represent the official position of the US
437 Centers for Disease Control and Prevention.

438

439

Confidential: For Review Only

References

1. Thomas LM, D'Ambruoso L, Balabanova D. Verbal autopsy in health policy and systems: a literature review. BMJ Glob Health. 2018;3(2):e000639.
2. World health statistics 2018: monitoring health for the SDGs, sustainable development goals. Geneva; 2018.
3. D'Ambruoso L, Kahn K, Wagner RG, Twine R, Spies B, van der Merwe M, et al. Moving from medical to health systems classifications of deaths: extending verbal autopsy to collect information on the circumstances of mortality. Glob Health Res Policy. 2016;1:2.
4. Fottrell E, Byass P. Verbal Autopsy: Methods in Transition. Epidemiologic Reviews. 2010;32(1):38-55.
5. Murray CJL, Lozano R, Flaxman AD, Serina P, Phillips D, Stewart A, et al. Using verbal autopsy to measure causes of death: the comparative performance of existing methods. BMC Medicine. 2014;12(1):5.
6. AbouZahr C. Verbal autopsy: who needs it? Population Health Metrics. 2011;9(1):19.
7. Thomas LM, D'Ambruoso L, Balabanova D. Use of verbal autopsy and social autopsy in humanitarian crises. BMJ Glob Health. 2018;3(3):e000640.
8. Verbal autopsy standards: the 2016 WHO verbal autopsy instrument [online]. 2017.
9. Soleman N, Chandramohan D, Shibuya K. Verbal autopsy: current practices and challenges. Bulletin of the World Health Organization. 2006;84(3):239 - 45.
10. Reeves BC, Quigley M. A review of data-derived methods for assigning causes of death from verbal autopsy data. International Journal of Epidemiology. 1997;26(5):1080-9.
11. Lerer LB, Butchart A, Blanche MT. 'A bothersome death'—Narrative accounts of infant mortality in Cape Town, South Africa. Social Science & Medicine. 1995;40(7):945-53.
12. King C, Zamawe C, Banda M, Bar-Zeev N, Beard J, Bird J, et al. The quality and diagnostic value of open narratives in verbal autopsy: a mixed-methods analysis of partnered interviews from Malawi. BMC Medical Research Methodology. 2016;16(1):13.
13. Gouda HN, Flaxman AD, Brolan CE, Joshi R, Riley ID, AbouZahr C, et al. New challenges for verbal autopsy: Considering the ethical and social implications of verbal autopsy methods in routine health information systems. Soc Sci Med. 2017;184:65-74.
14. Moyer CA, Johnson C, Kaselitz E, Aborigo R. Using social autopsy to understand maternal, newborn, and child mortality in low-resource settings: a systematic review of the literature. Glob Health Action. 2017;10(1):1413917.
15. Aborigo RA, Allotey P, Tindana P, Azongo D, Debpuur C. Cultural imperatives and the ethics of verbal autopsies in rural Ghana. Global Health Action. 2013;6(1):18570.
16. Gouda HN, Kelly-Hanku A, Wilson L, Maraga S, Riley ID. "Whenever they cry, I cry with them": Reciprocal relationships and the role of ethics in a verbal autopsy study in Papua New Guinea. Soc Sci Med. 2016;163:1-9.
17. Morrison J, Fottrell E, Budhatokhi B, Bird J, Basnet M, Manandhar M, et al. Applying a Public Health Ethics Framework to Consider Scaled-Up Verbal Autopsy and Verbal Autopsy with Immediate Disclosure of Cause of Death in Rural Nepal. Public Health Ethics. 2018;11(3):293-310.
18. Bird J, Byass P, Kahn K, Mee P, Fottrell E. A matter of life and death: practical and ethical constraints in the development of a mobile verbal autopsy tool. Proceedings of the SIGCHI Conference on Human Factors in Computing Systems; Paris, France: Association for Computing Machinery; 2013. p. 1489–98.
19. Rankin JC, Lorenz E, Neuhann F, Yé M, Sié A, Becher H, et al. Exploring the role narrative free-text plays in discrepancies between physician coding and the InterVA regarding determination of malaria as cause of death, in a malaria holo-endemic region. Malaria Journal. 2012;11(1):51.
20. Joshi R, Praveen D, Chow C, Neal B. Effects on the estimated cause-specific mortality fraction of providing physician reviewers with different formats of verbal autopsy data. Population Health Metrics. 2011;9(1):33.

Confidential: For Review Only

21. Bar-Zeev N, Kapanda L, King C, Beard J, Phiri T, Mvula H, et al. Methods and challenges in measuring the impact of national pneumococcal and rotavirus vaccine introduction on morbidity and mortality in Malawi. Vaccine. 2015;33(23):2637-45.

22. National Statistical Office/Malawi, ICF. Malawi Demographic and Health Survey 2015-16. Zomba, Malawi: National Statistical Office and ICF; 2017.

23. King C, Beard J, Crampin AC, Costello A, Mwansambo C, Cunliffe NA, et al. Methodological challenges in measuring vaccine effectiveness using population cohorts in low resource settings. Vaccine. 2015;33(38):4748-55.

24. Hartung C, Lerer A, Anokwa Y, Tseng C, Brunette W, Borriello G. Open data kit: tools to build information services for developing regions. Proceedings of the 4th ACM/IEEE International Conference on Information and Communication Technologies and Development; London, United Kingdom. 2369236: ACM; 2010. p. 1-12.

25. Verbal autopsy standards: the 2012 WHO verbal autopsy instrument [online]. Geneva; 2012.

26. BAIRAGI R, BLACK R, BLACKER J, BOERMA T, DALESSANDRO U, DAVID P, et al. MEASUREMENT OF OVERALL AND CAUSE-SPECIFIC MORTALITY IN INFANTS AND CHILDREN-MEMORANDUM FROM A WHO UNICEF MEETING (REPRINTED FROM BULLETIN DE LORGANISATION MONDIALE DE LA SANTE, VOL 72, PG 707-713, 1994). BULLETIN OF THE WORLD HEALTH ORGANIZATION. 1995;73(2):149-56.

27. Chandramohan D, Soleman N, Shibuya K, Porter J. Editorial: Ethical issues in the application of verbal autopsies in mortality surveillance systems. Tropical Medicine & International Health. 2005;10(11):1087-9.

28. Meyer AC, Opoku C, Gold KJ. "They Say I Should not Think About It:": A Qualitative Study Exploring the Experience of Infant Loss for Bereaved Mothers in Kumasi, Ghana. Omega (Westport). 2018;77(3):267-79.

29. Haws RA, Mashasi I, Mrisho M, Schellenberg JA, Darmstadt GL, Winch PJ. "These are not good things for other people to know": how rural Tanzanian women's experiences of pregnancy loss and early neonatal death may impact survey data quality. Soc Sci Med. 2010;71(10):1764-72.

30. Van Malderen C, Van Oyen H, Speybroeck N. Contributing determinants of overall and wealth-related inequality in under-5 mortality in 13 African countries. Journal of Epidemiology and Community Health. 2013;67(8):667-76.

31. Van Malderen C, Amouzou A, Barros AJD, Masquelier B, Van Oyen H, Speybroeck N. Socioeconomic factors contributing to under-five mortality in sub-Saharan Africa: a decomposition analysis. BMC Public Health. 2019;19(1):760.

32. Wijngaards-de Meij L, Stroebe M, Schut H, Stroebe W, van den Bout J, van der Heijden P, et al. Couples at Risk Following the Death of Their Child: Predictors of Grief Versus Depression. Journal of Consulting and Clinical Psychology. 2005;73(4):617-23.

33. Demmer C. Experiences of women who have lost young children to AIDS in KwaZulu-Natal, South Africa: a qualitative study. Journal of the International AIDS Society. 2010;13(1):50.

34. Smith H, Asfaw AG, Aung KM, Chikoti L, Mgawadere F, d'Aquino L, et al. Implementing the WHO integrated tool to assess quality of care for mothers, newborns and children: results and lessons learnt from five districts in Malawi. BMC Pregnancy and Childbirth. 2017;17(1):271.

35. Chou VB, Walker N, Kanyangarara M. Estimating the global impact of poor quality of care on maternal and neonatal outcomes in 81 low- and middle-income countries: A modeling study. PLoS medicine. 2019;16(12):e1002990-e.

36. Vanclay F, Baines JT, Taylor CN. Principles for ethical research involving humans: ethical professional practice in impact assessment Part I. Impact Assessment and Project Appraisal. 2013;31(4):243-53.

37. Wing DG, Burge-Callaway K, Rose Clance P, Armistead L. Understanding gender differences in bereavement following the death of an infant: Implications of or treatment. Psychotherapy: Theory, Research, Practice, Training. 2001;38(1):60-73.

Confidential: For Review Only

38. Treviño-Siller S, Ramírez-Villalobos D, Sanchez-Dominguez MS, Hernández Prado B. How to deal with the suffering: Utility of an emotional containment strategy to collect data for verbal autopsies in Mexico. Death Studies. 2020:1-6.
39. McNeil MJ, Namisango E, Hunt J, Powell RA, Baker JN. Grief and Bereavement in Parents After the Death of a Child in Low- and Middle-Income Countries. Children. 2020;7(5):39.

Confidential: For Review Only

Figure 1: Verbal autopsy inclusion (CONSORT diagram)

Table 1: Description of VA duration.

	No Narrative N = 1265	Narrative N = 1242	*p-value*
**Total VA duration (Minutes)**			
< 10	259 (20.5%)	33 (2.7%)	
11 – 20	663 (52.4%)	381 (30.7)	
21 – 30	252 (19.9%)	422 (34.0%)	
> 30	66 (5.2%)	401 (32.3%)	
Missing	25 (2.0%)	5 (0.4%)	<0.001
**Closed question duration (Minutes)***			
Min – Max	6 – 134	6 – 164	
Median (IQR)	15.0 (9.7)	19.8 (9.9)	<0.001
**Narrative duration (Minutes)**			
Min – Max		1 – 113	
Median (IQR)		7.0 (5.0)	

*The duration included pauses in the interview; MEOs were instructed to allow at least 30 minutes if the respondent needed to pause the interview before attempting to reschedule. Note, only 17 (0.7%) interviews took longer than 60 minutes.

Confidential: For Review Only

Table 2: Description of respondent emotional stress and VA quality indicators, between interviews with and without open narratives.

	No Narrative n (%)	Narrative n (%)	Total n (%)	*p-value*
**Respondent displayed visible emotional distress**				
No	1042 (82.4%)	990 (79.7%)	2032 (81.0%)	
Yes	223 (17.6%)	252 (20.3%)	475 (19.0%)	0.089
***Type of emotional distress displayed during interview**				
Crying	4 (1.8%)	12 (4.8%)	18 (3.4%)	
Long silence	59 (26.5%)	68 (27.0%)	127 (26.7%)	
Other signs of emotional distress	160 (71.8%)	172 (68.2%)	332 (69.9%)	0.191
***Interview paused due to respondent becoming emotionally distressed**				
No	89 (39.9%)	117 (46.4%)	206 (43.4%)	
Yes – Once	31 (13.9%)	41 (16.3%)	72 (15.2%)	
Yes – More than once	103 (46.2%)	94 (37.3%)	197 (41.5%)	0.146
**Respondent expressed desire to know the cause of death**				
No	1235 (97.6%)	1216 (97.9%)	2451 (97.8%)	
Yes	30 (2.4%)	26 (2.1%)	56 (2.2%)	0.638
**Respondent suggested potential cause of death**				
No	909 (71.9%)	890 (71.7%)	1799 (71.8%)	
Yes	356 (28.1%)	352 (28.3%)	708 (28.2%)	0.912
**Inter-VA able to assign cause of death**				
Indeterminate	77 (6.1%)	66 (5.3%)	143 (5.7%)	
Determinate	1188 (93.9%)	1176 (94.7%)	2364 (94.3%)	0.404
**Number of "Yes" responses to closed questions****	**Mean (SD)**			
Neonates	22.6 (5.3)	22.2 (5.3)	22.4 (5.3)	0.297
Infants	21.5 (6.8)	21.3 (7.3)	21.4 (7.0)	0.658
Child	22.2 (8.2)	21.3 (8.1)	21.8 (8.1)	0.122

*Questions only asked for respondents who had a visible display of emotional distress (n=475); "other" was not further specified, but informal feedback from MEOs reported examples of distress seen in facial expressions and body language.

**Different numbers of questions are asked for different age groups

Confidential: For Review Only

Table 3: Logistic regression exploring associations between respondent and child characteristics and emotional distress during VA.

Visible emotional distress due to open narrative			
**Descriptors**		**aOR* (95% CI)**	***p-value***
**Open narrative**	No	1.00	
	Yes	1.20 (0.98, 1.47)	0.084
**Respondent**	Mother	1.00	
	Father	0.72 (0.49, 1.07)	0.102
	Grandparent	0.23 (0.13, 0.39)	<0.001
	Others	0.04 (0.01, 0.28)	0.001
**Child's age**	Neonate	1.00	
	Infant	1.42 (1.09, 1.85)	0.010
	Child under-5	1.21 (0.86, 1.69)	0.274
**Child's sex**	Male	1.00	
	Female	0.99 (0.80, 1.22)	0.920
**Location of death**	Home	1.00	
	Health centre	1.36 (1.04, 1.77)	0.023
	MDH	0.96 (0.72, 1.27)	0.753
	En route to hospital	1.49 (1.00, 2.22)	0.049
	Other	0.38 (0.23, 0.64)	<0.001
**Socio-economic status by tercile**	Tercile 1 (Lowest)	1.00	
	Tercile 2 (Middle)	1.52 (1.17, 1.97)	0.002
	Tercile 3 (Highest)	1.49 (1.13, 1.95)	0.004
**Delay between death & VA (Weeks)**		0.98 (0.98, 0.99)	0.002

*All variables presented were included in the adjusted analysis

Confidential: For Review Only

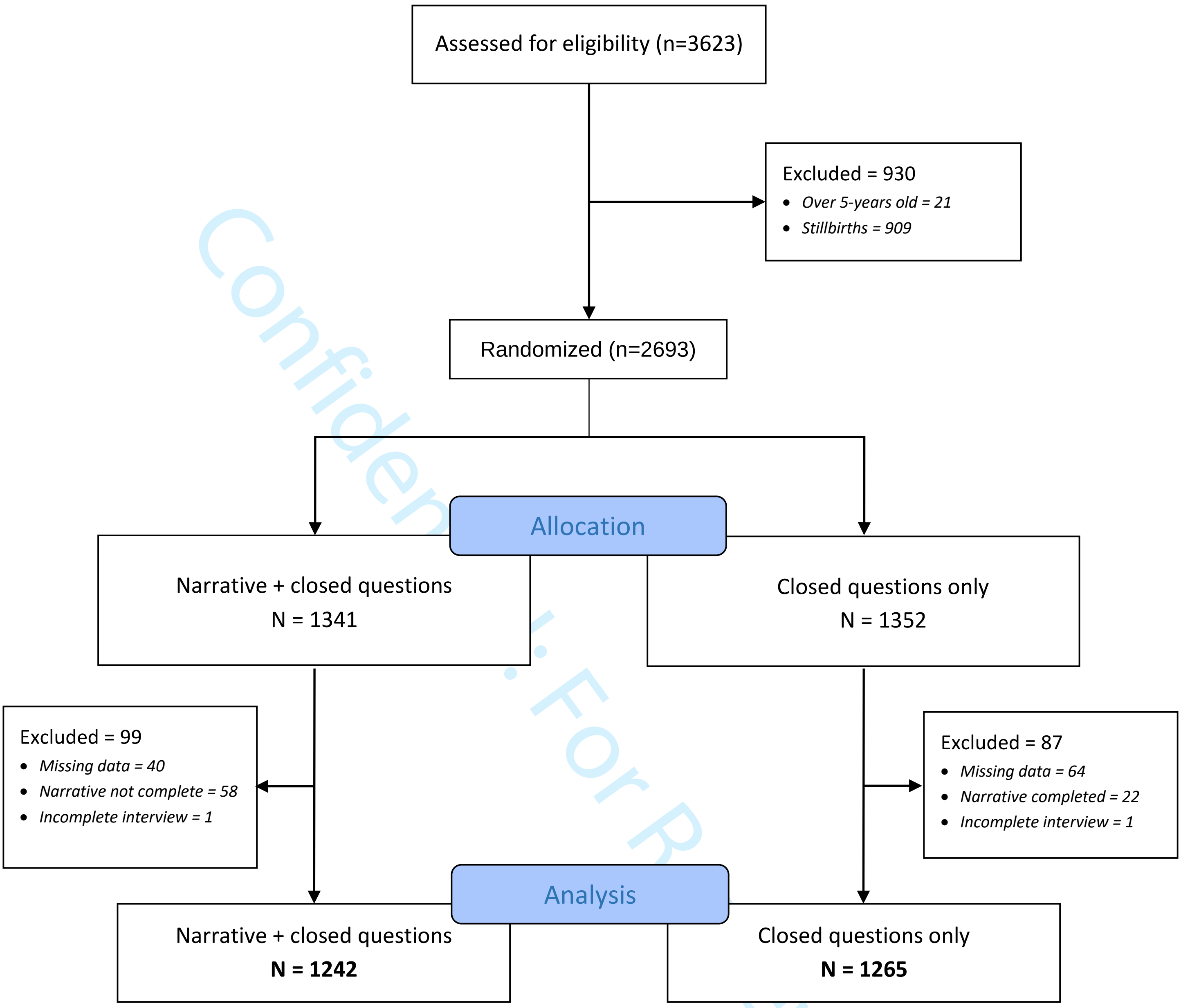

Confidential: For Review Only

Web-Appendix 1: Group discussion topic guide

1. What is your experience of conducting verbal autopsies?
2. Can you describe the typical interview process?
3. Thinking specifically about interviews which DID NOT have an open narrative:
   a. How did you establish a rapport with the respondents?
   b. Did you feel the quality of the responses was accurate?
   c. Were there any issues in conducting the interviews?
4. Now thinking specifically about the interviews which DID have an open narrative:
   a. How did you establish a rapport with the respondents?
   b. Did you feel the quality of the responses was accurate?
   c. Were there any issues in conducting the interviews?
5. Overall, what were the biggest challenges in conducting the verbal autopsies?
6. Overall, did you feel there were any positive aspects of conducting these interviews?
7. Did you feel the open narrative improved the interview process for respondents? And for you? Why / Why not?
8. If you could recommend a best practice for conducting verbal autopsies, what would it be? Why?

Confidential: For Review Only

Web-appendix 2: Comparison of interviews which did not comply with the randomisation protocol

	Interview conducted per protocol (n=2507)	Protocol violation	
		No narrative group (n=22)	Narrative group (n=58)
**Respondent**			
Mother	1931 (77.0%)	19 (86.4%)	46 (79.3%)
Father	200 (8.0%)	2 (9.1%)	4 (6.9%)
Grandparent	266 (10.6%)	-	6 (10.3%)
Other	110 (4.4%)	1 (4.6%)	2 (3.5%)
**Socio-economic status by tercile**			
Tercile 1 (Lowest)	838 (33.4%)	6 (27.3%)	20 (34.5%)
Tercile 2 (Middle)	879 (35.1%)	8 (36.4%)	19 (32.8%)
Tercile 3 (Highest)	790 (31.5%)	8 (36.4%)	19 (32.8%)
**Child's age**			
Neonate (0-4 weeks)	1058 (41.9%)	7 (32.8%)	19 (32.8%)
Infant (5-52 weeks)	654 (26.4%)	10 (45.5%)	18 (31.0%)
Child (12-59 months)	795 (31.7%)	5 (22.7%)	21 (36.2%)
**Child's sex***			
Male	1326 (52.9%)	9 (40.1%)	24 (42.1%)
Female	1181 (47.1%)	13 (59.1%)	33 (57.9%)
**Location of death**			
Home	797 (31.8%)	8 (36.4%)	13 (22.4%)
Health centre	652 (26.0%)	5 (22.7%)	20 (34.5%)
MDH	616 (24.6%)	5 (22.7%)	12 (20.7%)
En-route to hospital	181 (7.2%)	-	6 (10.3%)
Other	261 (10.4%)	4 (18.2%)	7 (12.1%)
	**Mean (SD)**		
Delay between death and VA (weeks)	22.5 (15.5)	25.5 (14.9)	23.0 (14.3)

*One child in the non-compliant narrative group was missing a value for sex.

Confidential: For Review Only

Web-Appendix 3: Comparison of randomised groups

	No Narrative N = 1265 (51%)	Narrative N = 1242 (49%)	Total N = 2507	*p-value*
**Respondent**				
Mother	985 (77.9%)	946 (76.2%)	1931 (77.0%)	
Father	95 (7.5%)	105 (8.5%)	200 (8.0%)	
Grandparent	125 (9.9%)	141 (11.4%)	266 (10.6%)	
Other	60 (4.7%)	50 (4.0%)	110 (4.4%)	0.400
**Socio-economic status by tercile**				
Tercile 1 (Lowest)	408 (32.3%)	430 (34.6%)	838 (33.4%)	
Tercile 2 (Middle)	459 (36.3%)	420 (33.8%)	879 (35.1%)	
Tercile 3 (Highest)	398 (31.5%)	392 (31.6%)	790 (31.5%)	0.343
**Child's age**				
Neonate (0-4 weeks)	505 (39.9%)	553 (44.5%)	1058 (41.9%)	
Infant (5-52 weeks)	352 (27.8%)	302 (24.3%)	654 (26.4%)	
Child (12-59 months)	408 (32.3%)	387 (31.2%)	795 (31.7%)	0.042
**Child's sex**				
Male	677 (53.5%)	649 (52.3%)	1326 (52.9%)	
Female	588 (46.5%)	593 (47.8%)	1181 (47.1%)	0.526
**Location of death**				
Home	408 (32.3%)	389 (31.3%)	797 (31.8%)	
Health centre	327 (26.9%)	325 (26.2%)	652 (26.0%)	
MDH	297 (23.5%)	319 (25.7%)	616 (24.6%)	
En route to hospital	78 (6.2%)	103 (8.3%)	181 (7.2%)	
Other	155 (12.3%)	106 (8.5%)	261 (10.4%)	0.008
	**Mean (SD)**	**Mean (SD)**		
Delay between death and VA (Weeks)	22.6 (15.5)	22.5 (15.4)	22.5 (15.5)	0.838

Confidential: For Review Only
